# The Association Between Psoriasis, Psoriatic Arthritis, and Fibromyalgia Syndrome: Effects on Treatment—A Population-Based Study

**DOI:** 10.3390/medicina61101809

**Published:** 2025-10-09

**Authors:** Yoav Elizur, Mor Amital, Niv Ben-Shabat, Chen Patt, Galia Zacay, Simon Lassman, Dennis McGonagle, Abdulla Watad, Omer Gendelman, Howard Amital

**Affiliations:** 1 Department of Internal Medicine B & Zabludowicz Center for Autoimmune Diseases, Sheba Medical Center, Ramat Gan 52621, Israel; yoav.elizur@mail.huji.ac.il (Y.E.); m.moramital@gmail.com (M.A.);; 2 Faculty of Medicine, Tel-Aviv University, Tel Aviv 69978, Israel; 3 The Adelson School of Medicine, Ariel University, Ariel 40700, Israel; 4 Department of Family Medicine, Meuhedet Health Maintenance Organization, Tel Aviv 62038, Israel; 5 St George’s Hospital, University of London, London SW17 0RE, UK; 6 Section of Musculoskeletal Disease, NIHR Leeds Musculoskeletal Biomedical Research Unit, University of Leeds, Chapel Allerton Hospital, Leeds LS7 4SA, UK

**Keywords:** psoriasis, fibromyalgia, psoriatic arthritis, spondyloarthritis, biologic therapy

## Abstract

*Background and Objectives:* To examine the prevalence of fibromyalgia syndrome (FMS) in patients with psoriasis (PsO) and psoriatic arthritis (PsA) and its impact on treatment patterns and biologic therapy adherence. *Materials and Methods:* This retrospective cohort study utilized electronic health records from the Meuhedet Health Maintenance Organization in Israel between 2000 and 2020. PsO patients were matched 1:4 with controls by age, sex, and ethnicity. We assessed FMS prevalence, comorbidity burden, and treatment patterns. Cox regression and linear models evaluated the association between FMS and biologic switching and duration, adjusting for confounders. *Results*: Among 61,003 PsO patients and 244,012 controls, FMS prevalence was higher in PsO (3.3% vs. 2.3%, OR = 1.45, 95% CI: 1.38–1.53, *p* < 0.001). Among PsO patients, those with FMS were predominantly female (81.1% vs. 49.8%, *p* < 0.001) and had a higher prevalence of PsA (33.6% vs. 7.7%, *p* < 0.001). They received biologics more frequently (10.2% vs. 2.7%, *p* < 0.001) and were more likely to require multiple biologic lines (4.2% vs. 0.7%, *p* < 0.001). In PsA patients receiving biologics, FMS was associated with reduced survival on first-line therapy (6.1 vs. 10.1 years), increased switching risk (HR = 1.82, 95% CI: 1.42–2.35), and shorter treatment duration (B= −0.97 years, *p* = 0.001). *Conclusions*: In PsO patients, especially those with psoriatic arthritis, FMS is linked to greater treatment complexity and shorter biologic therapy survival, underscoring the need for tailored management strategies.

## 1. Introduction

Psoriasis (PsO) is a chronic immune-mediated disease affecting 125 million people worldwide [1]. Although psoriasis is primarily a cutaneous disease, it often causes extracutaneous manifestations as well, mostly notably psoriatic arthritis (PsA), which affects up to a third of individuals with psoriasis and is associated with adverse clinical outcomes [2,3]. While PsA is considered a separate disease, it has significant comorbidity with PsO and typically compromises musculoskeletal structures as well as the eyes, digestive system, liver and kidneys [4]. It is also associated with a 43% increased risk of developing cardiovascular disease [5].

Previous studies have described the relationship between the fibromyalgia syndrome (FMS) and PsA and its adverse effect on functional capacity [6,7]. FMS prevalence rises in the context of rheumatic diseases [8], including rheumatoid arthritis, and is characterized by widespread pain, unrefreshing sleep, fatigue, and cognitive impairment with a significant female predominance [9]. Because of the overlap in symptoms, there is an increased burden of illness and a decline in quality of life [10,11].

It is particularly important to identify FMS in patients with PsO and to avoid misclassifying it as PsA, since FMS may be mistaken for inflammatory arthritis and lead to inappropriate use of DMARDs and biologic therapies [12]. Multisite enthesitis may serve as an essential steppingstone in the transition from psoriasis to PsA. Enthesitis is frequent in active PsA and, similar to FMS, is characterized by tenderness at multiple sites, absence of visible swelling, normal inflammatory markers, and the lack of gold-standard imaging or histopathological confirmation.

Moreover, psoriasis has been associated with a higher prevalence of depression among individuals compared to the general population, and there seems to be a correlation between the severity of the cutaneous disease and depressive symptoms [13]. A recent study conducted by Wong et al. [14] demonstrated that symptoms of anxiety and depression reduce the probability of achieving sustained minimal disease activity (MDA) in psoriatic arthritis. The study also found that the presence of fibromyalgia in PsA patients diminishes their likelihood of attaining MDA [14]. The pathophysiology of FMS is thought to be related to dysfunction in neurotransmitters including serotonin and glutamate, that may explain the increased association with somatic and psychiatric conditions, including greater susceptibility to experiencing depression [15].

This study aimed to describe the association between PsO and FMS and to focus on the comorbidity of these two disorders and their impact on adherence patterns with different therapeutic regimens. In particular, we investigated the rates of “treatment-switching” from one biologic to another, analyzing drug survival rates on the first line of biologics and comparing the mean duration of each biologic treatment.

## 2. Methods

### 2.1. Study Design and Setting

Electronic records from the Meuhedet Health Maintenance Organization (MHMO), which provides healthcare to approximately 1.2 million individuals in Israel, provided information on demographics, diagnostic codes, laboratory results, medication dispensations, and hospitalizations. These data were analyzed within a retrospective cohort design. Databases from Israeli HMOs, including Meuhedet, are widely recognized as reliable resources for population-based research and have supported numerous peer-reviewed publications [16,17,18].

The study followed participants from 1 January 2000 to 31 December 2020. Approval was obtained from the MHMO institutional ethics board. As this was a retrospective analysis of de-identified data, informed consent was waived.

### 2.2. Study Population

All individuals with a recorded diagnosis of psoriasis (ICD-9 code 696.*) during the study period were included in the cohort. For each psoriasis case, four control participants without psoriasis were randomly selected, matched on date of birth, sex, and ethnicity. Follow-up data were available through 10 February 2022.

### 2.3. Variables and Measures

Data for the analysis originated from the electronic medical records of the Meuhedet Health Maintenance Organization (MHMO). Baseline demographic variables, including age, sex, and ethnicity (classified as Arab or Jewish), were collected at the date of the first psoriasis diagnosis recorded in the database. The primary outcome was the presence of a documented diagnosis of fibromyalgia syndrome (FMS) [ICD-9 code 729.1]. We also obtained information on spondyloarthropathy-related comorbidities, including psoriatic arthritis [ICD-9 codes 696.0, 713.3], axial spondyloarthritis [ICD-9 codes 720, 720.0], and inflammatory bowel disease (Crohn’s disease and ulcerative colitis, ICD-9: 555.* and 556.*). For this study, psoriatic arthritis (PsA) was defined as the presence of psoriasis together with peripheral psoriatic arthritis, axial spondyloarthritis, or both.

Body mass index (BMI) was determined from the height and weight measurements closest to the index date of psoriasis diagnosis and classified as obese (≥30 kg/m^2^) or non-obese. Additional chronic conditions—including diabetes, ischemic heart disease (IHD), stroke, chronic obstructive pulmonary disease (COPD), congestive heart failure (CHF), malignancy, depression, hypertension (HTN), dyslipidemia, dementia, and smoking status—were identified through ICD-9 codes recorded in the MHMO database. Comorbidities were considered present if coded at any time during follow-up, whether before or after the initial psoriasis diagnosis.

Therapeutic exposures, including phototherapy, methotrexate, anti-TNF agents, anti-IL-12/23 agents, anti-IL-17 agents, and JAK inhibitors, were captured according to pharmacy dispensation data following psoriasis diagnosis. The duration of each treatment was calculated based on the recorded dates of first and last purchase.

### 2.4. Statistical Analysis

Continuous variables were summarized as mean ± standard deviation when normally distributed, or as median with interquartile range when non-normally distributed. Group comparisons were performed using Student’s *t*-test or the Mann–Whitney U test, as appropriate. Categorical variables were expressed as percentages and compared using Pearson’s chi-square test.

Both cross-sectional and longitudinal analyses were performed. Prevalence of comorbidities and treatment use was assessed descriptively, based on the presence of diagnostic codes at any time during follow-up. For treatment outcomes, we used the longitudinal structure of the dataset to analyze time to switching from first-line biologic therapy with Kaplan–Meier survival curves and Cox proportional hazards models. Multivariable linear regression was applied to evaluate the association between FMS and duration of biologic therapy, adjusting for age, sex, and ethnicity.

We calculated 95% confidence intervals (CIs) to estimate parameter precision and uncertainty. All *p*-values were two-tailed, and the alternative hypothesis was considered significant if *p* ≤ 0.05. Missing data were present in less than 5% of key variables and were handled using case-wise deletion without imputation. Statistical analyses were conducted using SPSS software, version 26 (Armonk, NY, USA: IBM Corp), and R Statistical Software, version 4.2.2.

## 3. Results

### 3.1. Study Population

The study comprised 61,003 patients with psoriasis and 244,012 age, sex, and ethnicity-matched controls (Table 1). The mean age at index was 36.1 years (SD ± 19.3 years), with equal distribution of females (50.8%) and Arab ethnicity (11.5%) across groups due to matching.

Psoriasis patients had a significantly higher prevalence of chronic comorbidities compared to the control group, including obesity (26.3% vs. 22.4%; *p* < 0.001), diabetes (16.3% vs. 14.0%; *p* < 0.001), HTN (29.9% vs. 27.4%, *p* < 0.001), IHD (10.2% vs. 9.0%; *p* < 0.001), stroke (5.9% vs. 5.4%; *p* < 0.001), COPD (9.5% vs. 7.8%; *p* < 0.001), CHF (3.0% vs. 2.6%; *p* < 0.001), dyslipidemia (45.0% vs. 41.1%, *p* < 0.001), and malignancy (11.9% vs. 10.5%; *p* < 0.001). Patients with psoriasis had a higher prevalence of psoriatic arthritis (8.6% vs. 2.3%, *p* < 0.001) and IBD (2.5% vs. 1.6%, *p* < 0.001).

The prevalence of fibromyalgia was significantly higher among patients with psoriasis compared to controls (3.3% vs. 2.3%, *p* < 0.001) with OR = 1.45 (95% CI 1.38–1.53).

Various treatments for psoriasis were used in our study group, with phototherapy being the most common (8.3%), followed by methotrexate (3.1%), anti-TNF agents (1.4%), anti-IL-12/23 agents (0.5%), JAK inhibitors (0.4%), and anti-IL-17 agents (0.3%). In addition, 0.9% of the patients utilized several biologic lines in their treatment regimen.

### 3.2. Characteristics of Patients with Psoriasis and Fibromyalgia

Out of 61,003 psoriasis patients, 2037 patients also had a diagnosis of FMS. The comparison between psoriatic patients with fibromyalgia and those without fibromyalgia is detailed in Table 2. Compared to psoriatic patients without FMS, the vast majority of the patients with FMS were females (81.1% vs. 49.8%, *p* < 0.001), with a higher dominance of Arab ethnicity (13.1% vs. 11.4%, *p* < 0.001). The mean age at psoriasis diagnosis was higher among FMS patients (46.0 ± 14.5 years vs. 36.3 ± 19.4 years, *p* < 0.001).

Patients with FMS had a significantly higher prevalence of obesity (38.1% vs. 25.8%, *p* < 0.001), diabetes (25.7% vs. 16%, *p* < 0.001), HTN (45% vs. 29.3%, *p* < 0.001), IHD (14.6% vs. 10%, *p* < 0.001), stroke (13.1% vs. 5.7%, *p* < 0.001), COPD (17.8% vs. 9.2%, *p* < 0.001), CHF (3.9% vs. 3.0%, *p* = 0.01), dyslipidemia (66.6% vs. 44.3%, *p* < 0.001), and malignancy (18.9% vs. 11.7%, *p* < 0.001). Patients with FMS also had a significantly higher prevalence of SpA-related comorbidities, with a 4-times higher prevalence of psoriatic arthritis (33.6% vs. 7.7%, *p* < 0.001) and a higher prevalence of IBD (5.8% vs. 2.3%, *p* < 0.001).

The prevalence of depression was 3-times higher among patients with FMS (43.2% vs. 13.8%, *p* < 0.001) with an odds ratio of 4.77 (95% CI: 4.35–5.22). The association between depression and FMS among psoriasis patients remained significant after adjusting for sex, ethnicity, age at psoriasis diagnosis, and all the described comorbidities (Obesity, Diabetes, HTN, IHD, stroke, COPD, CHF, dyslipidemia, malignancy, SpA-related comorbidities) (adjusted OR = 3.01, 95% CI: 2.72–3.33).

Patients with psoriasis and fibromyalgia received more treatments across all modalities, with significantly higher usage of biologics (10.2% vs. 2.7%, *p* < 0.001). The prevalence of usage of multiple biologics lines was six times higher among the FMS patients (4.2% vs. 0.7%, *p* < 0.001).

### 3.3. Patterns of Biologics Usage Among Patients with Psoriasis and Fibromyalgia

**Figure 1:**
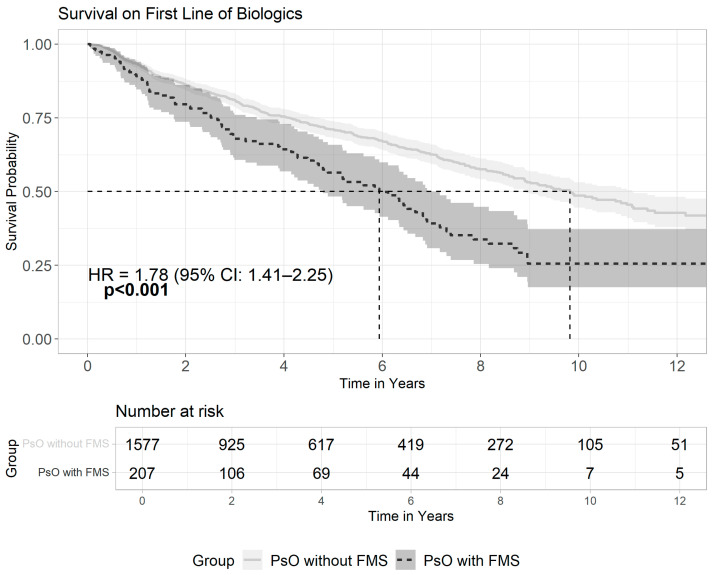
Survival on the first line of biologics among PsO patients with or without FMS. This Kaplan–Meier survival curve illustrates the time to switch from the first biologic treatment among psoriasis patients, comparing patients with FMS to patients without FMS. Psoriatic patients with co-existence of FMS exhibited significantly shorter drug survival (log-rank *p*-value < 0.001) and a higher risk for drug switching (HR = 1.78, 95% CI: 1.41–2.25). The solid line represents PsO patients without FMS, and the dashed line represents PsO patients with FMS. The vertical dashed lines denote the median time to switch: 5.9 years (FMS) vs. 9.8 years (without FMS). Shaded areas around each curve represent the 95% confidence intervals.

In our study, 1784 psoriatic patients were treated with biologics. Out of this group, 207 patients (11.6%) had a diagnosis of FMS, and these patients were more likely to receive multiple lines of biologics (41.1% vs. 27.9%, *p* < 0.001) with an odds ratio of 1.8 (95% CI: 1.34–2.43) (see Supplementary Table S1 and Supplementary Figure S1). Kaplan–Meier curve (Figure 1) demonstrates patients with the co-existence of PsO and FMS had significantly lower survival rates on the first line of biologic therapy (log-rank *p* < 0.001), with a median survival of 5.9 years (95% CI: 4.79–6.91 years) compared to 9.8 years in PsO patients without FMS (95% CI: 8.9–11.08 years). Using Cox (proportional hazards) regression model, the hazard for drug switching in the presence of FMS is multiplied by 1.78, and the effect is statistically significant (HR = 1.78, 95% CI: 1.41–2.25, *p* < 0.001).

**Figure 2:**
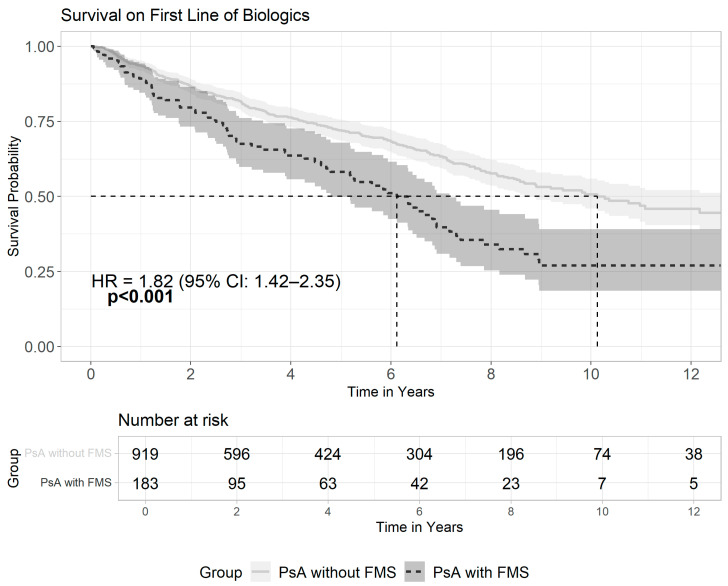
Survival on the first line of biologics among PsA patients with or without FMS. This Kaplan–Meier survival curve illustrates the time to switch from the first biologic treatment among psoriatic arthritis patients, comparing patients with FMS to patients without FMS. Patients with co-existence of FMS exhibited significantly shorter drug survival (log-rank *p*-value < 0.001) and a higher risk for drug switching (HR = 1.82, 95% CI: 1.42–2.35). The solid line represents PsA patients without FMS, and the dashed line represents PsA patients with FMS. The vertical dashed lines denote the median time to switch: 6.1 years (FMS) vs. 10.1 years (without FMS). Shaded areas around each curve represent the 95% confidence intervals.

Breaking down psoriatic patients who received biologics, 1102 of them (61.8%) had psoriatic arthritis. Out of the PsA patients, 183 patients (16.6%) had a diagnosis of FMS, and compared to patients without FMS, these patients were also more likely to receive multiple lines of biologics (42.1% vs. 30.6%, *p* < 0.001) with an odds ratio of 1.65 (95% CI: 1.19–2.28) (see Supplementary Table S2 and Supplementary Figure S2). Kaplan–Meier curve (Figure 2) demonstrates patients with the co-existence of FMS and PsA had significantly lower survival rates on the first line of biologic (log-rank *p* < 0.001), with a median survival of 6.1 years (95% CI: 4.79–7.17 years) compared to 10.1 years in PsA patients without FMS (95% CI: 8.68–16.3 years). The hazard for drug switching in FMS was 1.82 times greater, and the effect is statistically significant (HR = 1.82, 95% CI: 1.42–2.35, *p* < 0.001). The mean duration of each biologic therapy line was significantly shorter among patients with FMS (3.0 ± 3.1 years vs. 4.2 ± 3.6 years, *p* < 0.001). FMS was associated with almost a year shorter duration of therapy after adjusting for age, sex, and ethnicity using multiple linear regression (Table 3).

**Figure 3:**
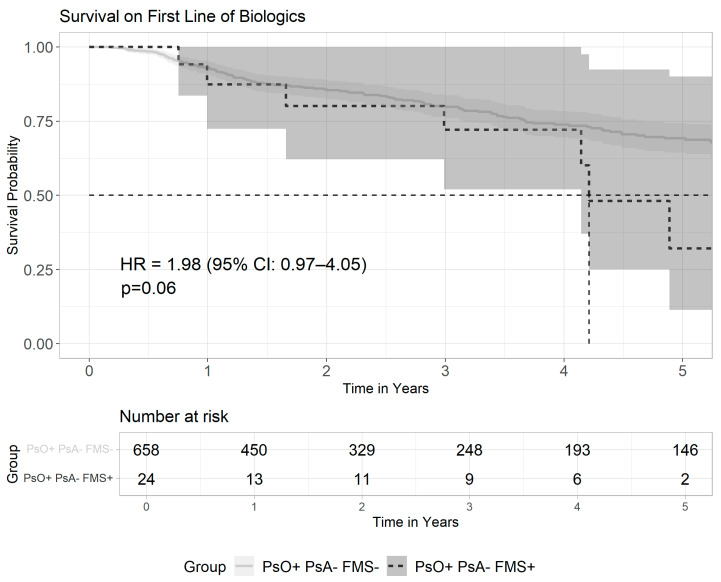
Survival on the first line of biologics among psoriatic patients without psoriatic arthritis with or without FMS. This Kaplan–Meier survival curve illustrates the time to switch from the first biologic treatment among psoriasis patients without arthritis involvement, comparing patients with FMS to patients without FMS. The differences between overall survival on first biologic and the risk for drug-switching were not statistically significant between the groups (log-rank *p* = 0.06; HR = 1.98, 95% CI: 0.97–4.05). The solid line represents patients without FMS, and the dashed line represents patients with FMS. The vertical dashed line denotes the median time to switch in the FMS group, while the median was not reached in the group without FMS. Shaded areas around each curve represent the 95% confidence intervals.

In the sub-group of 682 psoriatic patients who received biologics but without psoriatic arthritis involvement, the rates of multiple lines of treatment were not significantly different between PsO patients with FMS and PsO patients without FMS (33.3% vs. 24.2%, *p* = 0.305) (see Supplementary Table S3 and Supplementary Figure S3). Kaplan–Meier curve and Cox proportional hazard model within this sub-group (Figure 3) did not show a significant risk for drug-switching (HR = 1.98, 95% CI: 0.97–4.05, *p* = 0.06).

## 4. Discussion

This study used a large-scale population dataset to investigate the association between FMS, PsO, and PsA, as well as their respective epidemiological characteristics. Objective criteria to distinguish enthesitis from FMS remain sometimes elusive; therefore, there is clinical value in raising awareness of this potential misdiagnosis that might lead to mistreatment.

We found that patients with psoriasis had a higher prevalence of chronic comorbidities compared with controls, particularly obesity, ischemic heart disease, diabetes, hypertension, stroke, and congestive heart failure. These findings are consistent with previous reports linking psoriasis to elevated cardiometabolic risk [19,20,21]. Proposed mechanisms involve immune dysregulation, with cytokines and chemokines that act as central mediators of the IL-23/Th17 axis, contributing to systemic inflammation, atherosclerosis, and increased cardiometabolic disease burden [22,23].

The occurrence of FMS comorbidity with the diagnosis of psoriasis was 2037 out of 61,003, significantly higher than the control group (3.3% vs. 2.3%, *p* < 0.001, OR = 1.45). This aligns with global prevalence estimates for FMS, ranging from 0.4% to 9.3%, in a study review by Queiroz [24]. The prevalence of FMS among the control correlates with a recent study in Israel; 2.0% to 2.6% [25]. This strengthens the hypothesis that PsO diagnosis is a risk factor for developing FMS [26].

The association between autoimmune diseases and pain syndromes, such as FMS, has been widely discussed. Buskila et al. [27] found 57% and 24% of rheumatoid arthritis (RA) and PsA patients, respectively, met the diagnostic criteria for FMS. Another study showed that among 100 Behcet’s disease patients 18% had FMS comorbidity [28]. Dhir et al. [29] showed comorbid FMS in 15% of 200 RA patients, with greater disease severity, mood changes and functional disability, compared to 2.5% of the 200 controls.

Kridin et al. [30] examine the relationship between FMS and PsO in a large-scale population study. They discovered that PsO was more common among patients with FMS than in matched controls. When comparing FMS patients with concurrent PsO to those with only FMS, it was found that those with PsO were diagnosed with FMS at a significantly older age, had a higher average BMI, and reported a greater frequency of smoking. These results are consistent with our study, which also noted that patients with both FMS and psoriasis tended to develop psoriasis later in life.

Consistent with previous FMS studies, our study found a significant female predominance, with a ratio of approximately 9:1 [31]. One study of 1269 psoriasis patients showed that over 13% of psoriatic women met the criteria for FMS, in contrast to only 2% of men [32]. Additionally, 18% of participants (137 women and 93 men) reported musculoskeletal discomfort without meeting the FMS criteria. These results highlighted the importance of not missing diagnosis and treatment of FMS, particularly among female patients.

Psychiatric comorbidities are commonly associated with FMS. The prevalence of depression was three times higher among psoriatic patients with FMS. This stayed valid even after adjusting for sex, ethnicity, and age. According to the modified 2011 American College of Rheumatology (ACR) criteria for FMS [33] and their later revision in 2016 [34], depression is considered part of the Symptom Severity score (SS score). This refers to a subjective self-reporting of depressive symptoms rather than a clinical diagnosis. The presence of depression is associated with diminished quality of life in patients with FMS [35,36]. In a study conducted by our group, 42 male patients diagnosed with major depressive disorder (MDD) and an equal number of age-matched females with MDD, along with age-matched male and female healthy controls, were examined for comorbid FMS. The presence of FMS was found to correlate with reduced quality of life among individuals with depression. Additionally, female gender was identified as a risk factor for the development of FMS in MDD patients with clinical correlation to the severity of FMS [37].

Our research revealed that psoriatic patients with FMS received broader treatment modalities and were more likely to undergo biologic therapy. The use of multiple biologic lines in FMS patients was six times greater compared to psoriatic patients without FMS. Our study specifically focused on the subgroup of psoriatic patients with psoriatic arthritis and FMS, as PsA is often associated with FMS in the literature [7,38,39]. In our study, the prevalence of PsA was notably higher among psoriatic patients with FMS than those without FMS. The majority of psoriatic patients who received biologics in our study had PsA involvement. Arthritis involvement in psoriasis frequently requires long adherence to biologic therapy, and therefore we focused on this specific group and investigated adherence patterns. Our results indicated that patients with PsO and comorbid FMS had lower survival rates on the first line of biologic therapy than those with PsO without FMS, and these effects were also observed in the sub-group of patients with PsA involvement. Using Cox regression analysis, we estimated that the risk of switching from the first biologic therapy to another is almost double in psoriatic patients with PsA who also have FMS. Interestingly, these findings were not significant in the sub-group of psoriatic patients without PsA involvement, possibly highlighting joint involvement as a crucial predictor of adherence to biologics. It should be noted, however, that the power may be too low to yield significant findings due to the small sample size in this subgroup.

Our results align with a recent study by Iannone et al. [40], which assessed 238 PsA patients undergoing biologic treatment, 58 of whom had comorbid FMS. This study found that PsA patients with FMS had substantially lower survival rates on biologic treatment, with only 50% continuing therapy beyond 32 months.

It should be noted that our dataset comprised patients diagnosed with PsO as early as 2000. This accounts for the relatively extended drug-survival periods in our study, as a considerable number of patients during that period were treated solely with anti-TNF therapy. The options for switching therapies were restricted due to the lack of alternative therapeutic choices at the time.

Dobkin et al. [41] found patient adherence to FMS treatment among females was associated with increased affective pain and decreased psychological distress in patients. In a previous study conducted by our group, which examined persistence and adherence among 3932 FMS patients, only 45% of patients received at least one medication in the year following diagnosis, and merely 28.8% had prescriptions filled twice within the first year. Overall, only 9.3% of patients demonstrated high adherence to therapy [42]. These results, combined with our current findings, emphasize the considerably low adherence rates among individuals with FMS, potentially leading to difficulties in medication compliance and frequent changes in medications.

Prikhodkina et al. [43] noted low treatment adherence among females with FMS (35.2%), while only 13.5% reported high adherence, attributing this to depression influencing communication between patients and healthcare providers.

Treatment of PsA with biologics may fail due to an inadequate response, loss of efficacy, or adverse effects. There is no clear definition of “treatment failure” for PsA patients, leaving clinicians unsure when to switch to alternative treatment. This is a vexing issue as pain in PsO or PsA could have an enthesitis component that is difficult to evaluate clinically.

This study has several limitations inherent to retrospective real-world analyses using administrative data.

First, diagnoses were based on administrative coding and were not validated against clinical criteria. Specifically, PsA diagnoses were not confirmed using CASPAR criteria, and FMS diagnoses lacked standardized symptom severity scoring or formal rheumatologic evaluation. In Israel, FMS diagnoses in health maintenance organizations (HMOs), including Meuhedet, are typically made by rheumatologists and recorded under ICD-9 code 729.1. Nonetheless, we recognize the potential for diagnostic misclassification, both for FMS and for PsA, particularly given overlapping symptomatology such as widespread pain or enthesitis. Although Meuhedet’s longitudinal database includes rich demographic and treatment data, it lacks detailed information on disease severity (e.g., joint counts, PASI scores), pain scores, or imaging findings. This limited our ability to distinguish between FMS-related symptoms and PsA disease activity.

Second, the definition of axial spondyloarthritis (ICD-9 codes 720 and 720.0) as psoriatic arthritis may sometimes be incorrect. However, we chose to include these codes, alongside the primary code for psoriatic arthropathy (ICD-9 code 696.0), as patients with co-existence of psoriasis and axial spondyloarthritis commonly fall into the category of psoriatic arthritis. The observed prevalence of axial spondyloarthritis among the control group was higher than typically reported in population-based studies [44]. This may reflect the use of axial spondylitis diagnostic codes by physicians in routine community practice, possibly without rheumatologic confirmation. While some degree of overdiagnosis is possible, we believe this pattern is consistent across both study groups and is unlikely to introduce differential misclassification.

Finally, although the study spans two decades, changes in biologic availability and prescribing practices over time may have influenced drug survival estimates. The relatively low proportion of PsO patients receiving biologic or targeted small-molecule therapies can be attributed to the inclusion of patients diagnosed in the early 2000s, when such treatments were less accessible and less commonly prescribed.

Despite these limitations, this study’s strengths include its large sample size and a population-based design. The results highlight the need for careful clinical assessment to distinguish FMS from active PsA and avoid unnecessary escalation of immunosuppressive therapy.

In conclusion, our results reaffirm the association between psoriatic disease, especially PsA, and FMS. Joint involvement and depression may be predictors of poor adherence to biologics, and coexisting FMS negatively affects adherence to drug therapy. Clinicians may use these findings to address low adherence risk among FMS patients before prescribing biologics and switching to other treatment lines.

## Figures and Tables

**Table 1:** Characteristics of the study population.

Characteristics	Psoriasis (n = 61,003)	Controls (n = 244,012)	*p*-Value
Demographics			
Age, mean ± SD	36.1 ± 19.3	36.1 ± 19.3	matched
Female gender, n (%)	31,008 (50.8)	124,003 (50.8)	matched
Arab ethnicity, n (%)	6996 (11.5)	27,984 (11.5)	matched
Chronic Comorbidities ^†^			
Obesity, n (%)	14,540 (26.3)	49,526 (22.4)	**<0.001**
Diabetes, n (%)	9937 (16.3)	34,065 (14.0)	**<0.001**
HTN, n (%)	18,213 (29.9)	66,802 (27.4)	**<0.001**
IHD, n (%)	6196 (10.2)	22,072 (9.0)	**<0.001**
Stroke, n (%)	3623 (5.9)	13,236 (5.4)	**<0.001**
COPD, n (%)	5808 (9.5%)	19,040 (7.8)	**<0.001**
CHF, n (%)	1822 (3.0)	6372 (2.6)	**<0.001**
Dyslipidemia, n (%)	27,460 (45.0)	100,389 (41.1)	**<0.001**
Malignancy, n (%)	7250 (11.9)	25,663 (10.5)	**<0.001**
Dementia, n (%)	1051 (1.7)	4757 (1.9)	**<0.001**
Depression, n (%)	9008 (14.8)	32,156 (13.2)	**<0.001**
Fibromyalgia, n (%)	2037 (3.3)	5678 (2.3)	**<0.001**
Smoking, n (%)	3402 (19.9)	12,869 (20)	0.6
SpA-related Comorbidities ^†^			
Psoriatic arthritis ^‡^, n (%)	5223 (8.6)	5637 (2.3)	**<0.001**
Peripheral arthritis, n (%)	3697 (6.1)	108 (0.0)	**<0.001**
Axial spondyloarthritis, n (%)	1958 (3.2)	5539 (2.3)	**<0.001**
Inflammatory Bowel Disease, n (%)	1495 (2.5)	3834 (1.6)	**<0.001**
Treatment			
Phototherapy, n (%)	5039 (8.3)	-	-
Methotrexate, n (%)	2289 (3.8)	-	-
Anti-TNF, n (%)	1385	-	-
Anti-IL-12/23, n (%)	247	-	-
Anti-IL-17, n (%)	128	-	-
JAKi, n (%)	24	-	-
Multiple biologics lines, n (%)	525 (0.9)	-	-

^†^ Chi-square test; ^‡^ Peripheral and axial spondyloarthritis are considered non-mutually exclusive subtypes of psoriatic arthritis; patients may be included in both. Boldface type indicates *p* < 0.05; SD = Standard deviation.

**Table 2:** Characteristics of PsO patients with and without FMS.

Characteristics (N = 61,003)	FMS (n = 2037)	Without FMS (n = 58,966)	*p*-Value
Demographics			
Age at psoriasis diagnosis, mean ± SD ^†^	46.0 ± 14.5	36.3 ± 19.4	**<0.001**
Female gender, n (%) ^‡^	1653 (81.1)	29,355 (49.8)	**<0.001**
Arab ethnicity, n (%) ^‡^	267 (13.1)	6729 (11.4)	**<0.001**
Chronic Comorbidities ^‡^			
Obesity, n (%)	767 (38.1)	13,773 (25.8)	**<0.001**
Diabetes, n (%)	524 (25.7)	9413 (16.0)	**<0.001**
HTN, n (%)	916 (45.0)	17,297 (29.3)	**<0.001**
IHD, n (%)	298 (14.6)	5898 (10.0)	**<0.001**
Stroke, n (%)	266 (13.1)	3357 (5.7)	**<0.001**
COPD, n (%)	363 (17.8)	5445 (9.2)	**<0.001**
CHF, n (%)	80 (3.9)	1742 (3.0)	**0.01**
Dyslipidemia, n (%)	1356 (66.6)	26,104 (44.3)	**<0.001**
Malignancy, n (%)	379 (18.9)	6871 (11.7)	**<0.001**
Depression, n (%)	881 (43.2)	8127 (13.8)	**<0.001**
Dementia, n (%)	46 (2.3)	1005 (1.7)	0.06
Smoking, n (%)	173 (22.2)	3229 (19.7)	0.09
SpA-related Comorbidities ^‡^			
Psoriatic arthritis, n (%):	685 (33.6)	4538 (7.7)	**<0.001**
Peripheral arthritis, n (%)	507 (24.9)	3190 (5.4)	**<0.001**
Axial spondyloarthritis, n (%)	284 (13.9)	1674 (2.8)	**<0.001**
Inflammatory bowel disease, n (%)	119 (5.8)	1376 (2.3)	**<0.001**
Treatment ^‡^			
Phototherapy, n (%)	192 (9.4)	4847 (8.2)	**<0.001**
Methotrexate, n (%)	304 (14.9)	1985 (3.4)	**<0.001**
Anti-TNF, n (%)	184 (9)	1226 (2.1)	**<0.001**
Anti-IL-12/23, n (%)	38 (1.9)	477 (0.8)	**<0.001**
Anti-IL-17, n (%)	66 (3.2)	343 (0.6)	**<0.001**
JAKi, n (%)	30 (1.5)	82 (0.1)	**<0.001**
Any biologics	207 (10.2)	1577 (2.7)	**<0.001**
Multiple biologics lines, n (%)	85 (4.2)	440 (0.7)	**<0.001**

^†^ Student’s *t*-test; ^‡^ Chi-square test; Boldface type indicates *p* < 0.05; SD = Standard deviation.

**Table 3:** Association Between Fibromyalgia and Mean Duration of Biologic Therapy (Years) in PsA: Multiple Linear Regression.

Variable	Group	Mean ± SD	B	Beta	95% CI	Pv
Fibromyalgia	No	4.2 ± 3.6	Ref.		−1.53 to −0.4	**0.001**
	Yes	3.0 ± 3.1	−0.97	−0.102		
Ethnicity	Jewish	4.2 ± 3.6	Ref.		−1.98 to −0.6	**<0.001**
	Arab	2.9 ±2.8	−1.29	−0.11		
Sex	Male	4.4 ± 3.5	Ref.		−0.94 to −0.10	**0.016**
	Female	3.7 ± 3.5	−0.51	−0.073		
Age at 1st Biologic Treatment			−0.02	−0.094	−0.04 to 0.01	**0.002**

Note: Boldface type indicates *p* < 0.05, SD = Standard deviation; CI = Confidence Interval; Pv = *p*-value; Ref. = reference.

## Data Availability

The raw data supporting the conclusions of this article will be made available by the authors on request.

## Supplementary Materials

The following supporting information can be downloaded at: https://www.mdpi.com/article/10.3390/medicina61101809/s1, Table S1: The association between FMS and multiple lines of biologics among psoriasis patients; Figure S1: Number of treatment lines of biologics therapy among psoriasis patients; Table S2: The association between FMS and multiple lines of biologics among PsA patients; Figure S2: Number of treatment lines of biologics among PsA patients with or without FMS; Table S3: The association between FMS and multiple lines of biologics among psoriatic patients without psoriatic arthritis; Figure S3: Number of treatment lines of biologics among psoriatic patients without psoriatic arthritis with or without FMS.
