# Supplementary material for: The Association Between Psoriasis, Psoriatic Arthritis, and Fibromyalgia Syndrome: Effects on Treatment—A Population-Based Study
Source: Medicina (Kaunas). 2025 Oct 9;61(10):1809. doi: 10.3390/medicina61101809 (PMC12566094; doi:10.3390/medicina61101809)

Supplementary Materials:

Table S1. The association between FMS and multiple lines of biologics among psoriasis patients.

**Number of Treatment Lines of Biologic Therapy**	**PsO and FMS treated with Biologics (n=207)**	**PsO without FMS treated with Biologics (n=1577)**	**p-value**
1 Line of Biologics, n (%)	122 (58.9)	1137 (72.1)	**<0.001**
Multiple Lines of Biologics, n (%)	**85 (41.1)**	**440 (27.9)**	**<0.001**
2 Lines of Biologics, n (%)	61 (29.5)	340 (21.6)	
3≤Lines of Biologics, n (%)	24 (11.6)	100 (6.3)	

Figure S1. Number of treatment lines of biologics therapy among psoriasis patients.

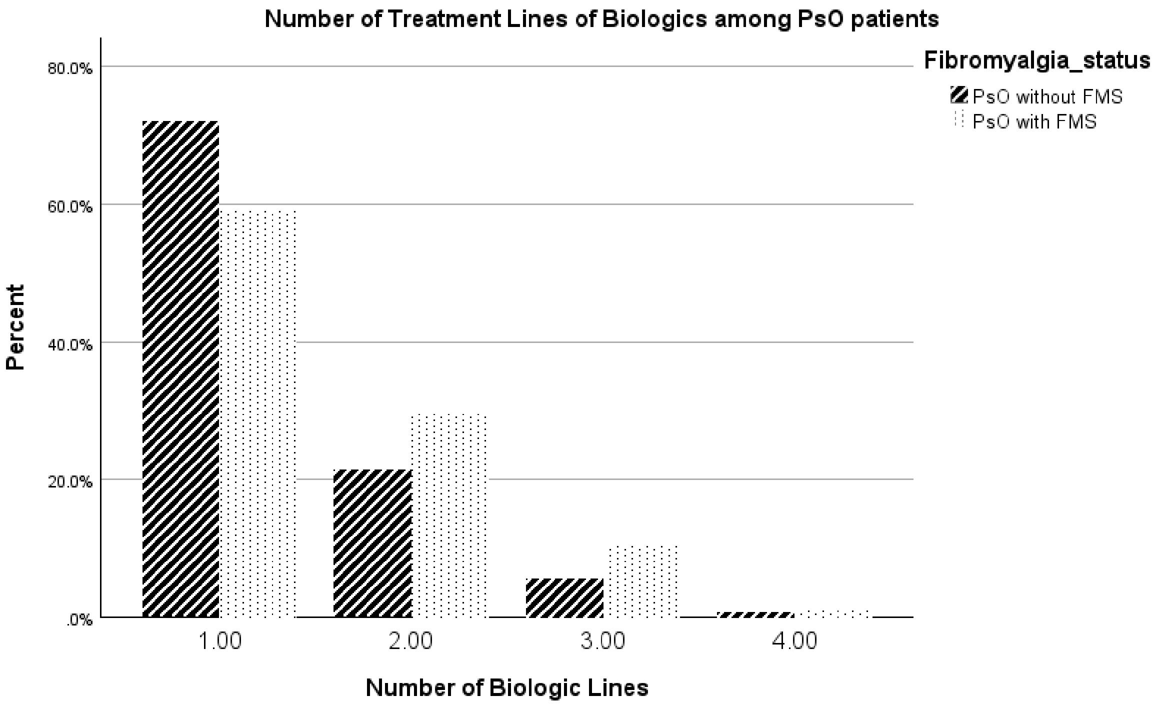

Table S2. The association between FMS and multiple lines of biologics among PsA patients.

**Number of Treatment Lines of Biologics**	**PsA and FMS treated with Biologics (n=183)**	**PsA without FMS treated with Biologics (n=919)**	**p-value**
Single Line of Biologics, n (%)	106 (57.9)	638 (69.4)	**<0.001**
Multiple Lines of Biologics, n (%)	**77 (42.1)**	**281 (30.6)**	**<0.001**
2 Lines of Biologics, n (%)	53 (29.0)	200 (21.8)	
3≤ Lines of Biologics, n (%)	24 (13.1)	81 (8.8)	

Figure S2. Number of treatment lines of biologics among PsA patients with or without FMS.

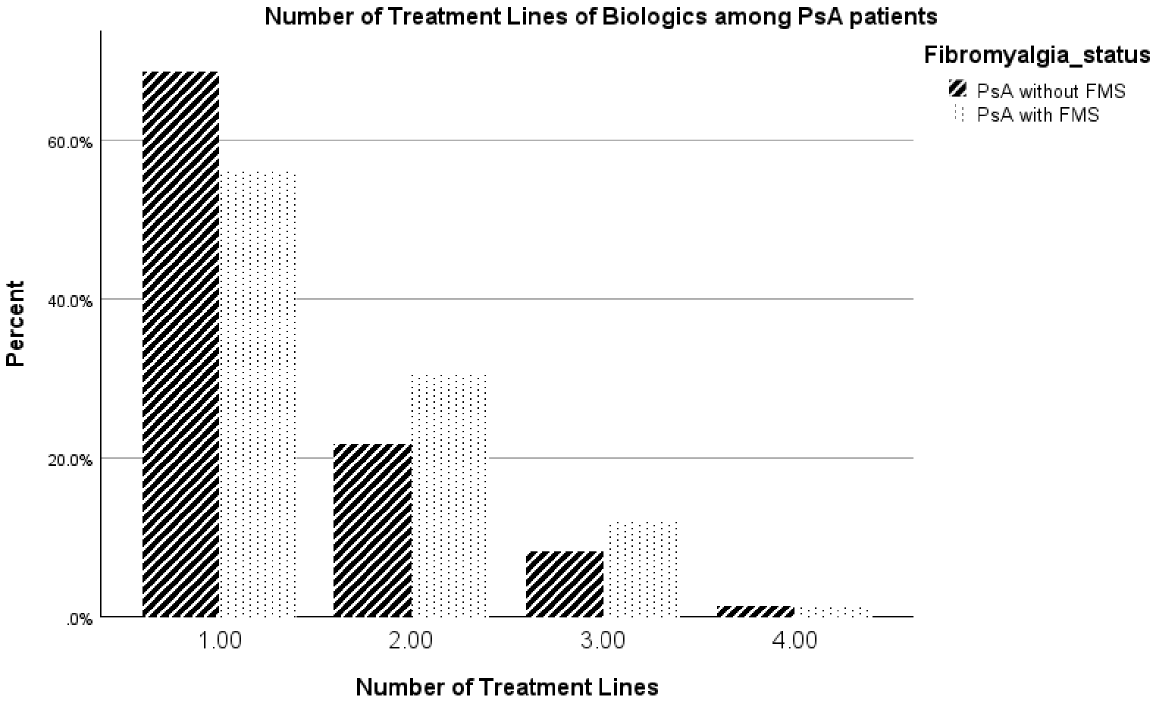

Table S3. The association between FMS and multiple lines of biologics among psoriatic patients without psoriatic arthritis.

**Number of Treatment Lines of Biologic Therapy**	**PsO+ PsA- FMS+ treated with Biologics (n=24)**	**PsO+ PsA- FMS- treated with Biologics (n=658)**	**p-value**
1 Line of Biologics, n (%)	16 (66.7)	499 (75.8)	0.305
Multiple Lines of Biologics, n (%)	8 (33.3)	159 (24.2)	

Figure S3. Number of treatment lines of biologics among psoriatic patients without psoriatic arthritis with or without FMS.

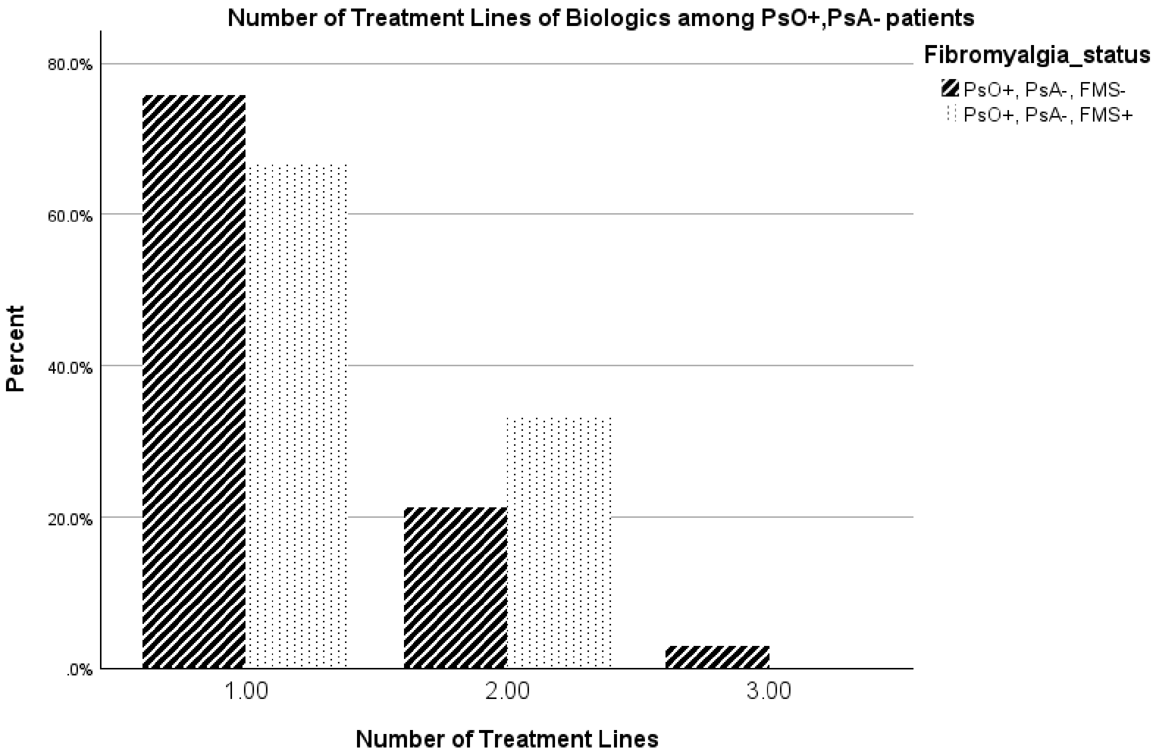

Supplement: Supplementary file 1 [file medicina-61-01809-s001.zip › medicina-3834081-supplementary.pdf]
